# Social networks respond to a disease challenge in calves

**DOI:** 10.1038/s41598-022-13088-2

**Published:** 2022-06-01

**Authors:** Katharine C. Burke, Sarah do Nascimento-Emond, Catherine L. Hixson, Emily K. Miller-Cushon

**Affiliations:** Department of Animal Sciences, University of Florida, Gainesville, FL 32611 USA

**Keywords:** Animal behaviour, Animal disease models

## Abstract

Changes in network position and behavioral interactions have been linked with infectious disease in social animals. Here, we investigate the effects of an experimental disease challenge on social network centrality of group-housed Holstein bull dairy calves. Within group-housed pens (6/group) calves were randomly assigned to either a previously developed challenge model, involving inoculation with *Mannheimia haemolytia* (*n* = 12 calves; 3 calves/group) or a control involving only saline (*n* = 12 calves; 3 calves/group). Continuous behavioral data were recorded from video on pre-treatment baseline day and for 24 h following inoculation to describe social lying frequency and duration and all active social contact between calves. Mixed-model analysis revealed that changes in network position were related to the challenge. Compared to controls, challenged calves had reduced centrality and connectedness, baseline to challenge day. On challenge day, challenged calves were less central in the directed social contact networks (lower degree, strength and eigenvector centrality), and initiated contact (higher out-degree) with more penmates, compared to healthy calves. This finding suggests that giving rather than receiving affiliative social contact may be more beneficial for challenged calves. This is the first study demonstrating that changes in social network position coincide with an experimental challenge of a respiratory pathogen in calves.

## Introduction

The identification and treatment of respiratory illness is a key challenge and major welfare concern for dairy calves^1^, prompting interest in behavioral changes coinciding with disease. Group-housed calves present an opportunity to understand changes in social networks and how they correspond to health. Studies of sickness behavior and the impact on social interaction patterns and network position have proven useful for wildlife disease ecology (e.g., vampire bats, *Desmodus rotundus*^2^; Tasmanian devils, *Sarcophilus harrisii*^3^). However, links between social networks and health are lacking for managed populations. In particular, there is limited understanding of how social relationships of gregarious livestock species may be related to health status.

Animals often respond to infection with cytokine-induced sickness behavior, which is considered to be a motivational state and includes fatigue, loss of appetite and social withdrawal^4^. Changes in social behavior have been characterized as a component of sickness behavior across group-living species. Both a reduction and increase in interactions have been observed; for example, wild mice (*Mus musculus domesticus)* limit the size of their social contact network in response to an infection^5^. Whereas in rhesus macaques (*Macaca mulatta*), increases in affiliative interactions with familiar peers and kin have been reported following an experimentally induced infection^6^. Humans have also been shown to both increase^7^ and limit^8–10^ their social interactions while sick, depending on the context and availability of social partners. In dairy calves, we found previously that individual calves initiated less social grooming following a respiratory disease challenge^11^, suggesting disease may be associated with changes in social relationships in group-housed dairy calves. In dairy calves, it is established that lying behavior is sensitive to naturally occurring illness, even increasing prior to clinical signs of illness^12^. However, no research to date has examined pairwise social interactions at the group level and consequently the social networks of dairy calves following infection.

Network approaches are valuable because they examine indirect connections beyond the level of the dyad and offer novel ways to uncover association between social behavior and animal health/welfare. Social network analysis (SNA) is a quantitative framework that is used to measure and analyze the patterns of individual and group level social interactions^13^. While it has found wide-ranging applications in animal behavior, only a handful of empirical studies to date have focused on calves^14–16^. Cattle form preferential social bonds and maintain affiliative relationships (e.g.,^17,18^). There are also indications that social relationships are important for calves. Research has shown that dairy calves benefit from group housing^19^ and exhibit social behaviors that are not found with individually housed calves^20,21^. For example, weaned dairy calves spend more time grooming a familiar companion compared with an unfamiliar one^22^. Further, studies of cattle in naturalistic settings suggest that young dairy calves rest within social groups (‘creches’) with calves of the same age^54^, indicating that social rest within same-age groups is an important natural behavior, with a speculated function of forming and reinforcing social bonds.

In this initial study, we quantified social networks for four groups of Holstein bull calves based on interaction data from a two-day experimental challenge that induced a component of bovine respiratory disease. Continuous behavioral data were recorded with digital video recording software. Social network position was measured using degree, strength and eigenvector centrality. We first establish whether social connections differ over the two-day challenge by comparing the consistency of dyadic social interactions and individual centrality scores. Next, to determine the possible effects of the experimental challenge on social network position, we examined individual centrality scores on challenge day and its relation to health status. Secondly, we tested whether an individual’s social network position differed significantly from baseline to challenge day and if this was related to the challenge. We hypothesize that the experimental challenge will result in reduced centrality and social connectedness for challenged calves, whereas control calves would maintain or increase connectedness with penmates.

## Methods

### Animal management

At the University of Florida Dairy Unit (Hague, FL, USA), Holstein bull calves (*n* = 24; aged 3 to 7 weeks) were placed into 4 groups based on age (6 calves/pen; 6.6 m^2^/calf) the week prior to the experiment. This was a convenience sample, with age range dependent upon the calf birth rate at the research facility. The age range within each pen was 7.5 ± 3.3 days (mean ± SE). This study was conducted prior to milk-weaning and calves were given pasteurized waste milk mixed with a powdered enhancer via teats, provided at a rate of 8 L/d in 2 daily meals (0600 and 1700 h). In addition, grain concentrate (via mounted buckets), and water were provided ad libitum.

### Experimental design and disease challenge

Calves were blocked into pairs by age and body weight and randomly assigned within pen to 1 of 2 treatments: (1) a disease challenge involving inoculation at the tracheal bifurcation with 3 × 10^9^ cfu of *Mannheimia haemolytica* (MH)*,* a main component of bovine respiratory disease^23^, suspended in 5 ml of sterile phosphate buffered saline followed by a 120 ml wash (*n* = 12 calves), or (2) control involving inoculation with phosphate buffered saline only (*n* = 12 calves)^24^. Treatments were imposed when the mean calf age (across all 4 pens) was 35.9 ± 8.8 days (mean ± SD). This disease challenge model was validated in 8-week-old dairy calves^25^. Based upon this data, a sample size of 8 was determined to be sufficient in detecting differences in lying behavior, using a level of significance of 0.05 and 80% power. However, we increased the sample size to 12 calves/treatment to increase power to detect differences in social interactions, which may be subject to greater individual variability than lying time. Effects of this disease challenge model on clinical health outcomes as well as some individual behavioral responses of calves in the present study were described previously^11^. Briefly, the experimental challenge caused a mild disease state: rectal temperatures of challenged calves were elevated, differing at 12 h post-inoculation (40.1 vs. 39.1; standard error = 0.14), and some changes in behavior including differences in lying laterality and decreased frequency of lying bouts^11^. The Institutional Animal Care and Use Committee (IACUC) of the University of Florida approved and deemed all procedures and experiments listed in this study were ethical for animal care (#201408643). In addition, the research was conducted in compliance with the ARRIVE guidelines, and all methods were performed in accordance with relevant guidelines and regulations.

### Behavioral observation

Video was recorded continuously throughout the trial (using Behavioral Observation Research Interactive Software; BORIS^26^). The average temperature during the study period was 25 degrees Celsius (min 23, max 31). Two 8 h. observational periods were conducted at 1100 h. on the day preceding treatment (baseline day; Day −1) and at 1100 h. immediately following inoculation (challenge day; Day 0). This window of observation was selected to evaluate the short-term acute response to the disease challenge, as previous data suggested that calf clinical signs of illness following this challenge were short-lived and returned to baseline within 24 h^25^. Individual calves were identified through coat markings and behavior (Table 1) was coded by one observer, who was blind to which calf underwent the disease challenge. Intra-observer reliability was calculated for one 8 h. period for 6 calves, with Cohen’s kappa = 0.94 as calculated in BORIS. The following behaviors were recorded continuously: frequency of social contact between individual calves and frequency and duration of social lying, with identity of all pairs of calves recorded. To further assess the direction of social interactions, initiated and received social contacts between calves were characterized on Day 0; these included all instances of directed behavior and excluded behaviors without a clear actor/recipient (such as side-to-side contact while passing). Social contacts and social lying were both observed to characterize different aspects of social behavior, including active social interactions, all physical social contact, and more passive social rest.

**Table 1:** Ethogram used to describe behaviors of calves (35.9 ± 8.8 days of age) housed in groups (6 calves/pen) exposed to either an experimental challenge model (inoculation with *M. haemolytica*) or a control (inoculation with sterile saline).

Measure	Behavior	Description
*Social lying (undirected)*	Social lying	Lying down within one body length of another calf, in any orientation; frequency and duration
Baseline and challenge day	–	–
*All-social contacts (undirected)*	Social contact	Any physical touch between calves in any posture and orientation; frequency
Baseline and challenge day	–	–
*Social contact (directed)*	Head butting	Pushing the head against the head or body of another calf; identified as actor or recipient
Challenge day only	Social grooming	Licking the head, neck, or body of another calf; identified as actor or recipient

### Social network data

Adjacency matrices were created from the frequency and duration of social interactions between calves in their respective pens. All networks had the same number of individual calves. A directed network was constructed from the frequency of all initiated social contacts, excluding any mutual contacts without clear actor/recipient on day of challenge only. Because this is a novel study aiming to investigate possible changes in social network position in response to an experimentally induced challenge, we opted to look at directed behaviors on challenge day only, since we were interested in the direction of affiliative behaviors. Additionally, three undirected networks were constructed with the frequency of social lying bouts, the duration of lying bouts, and the frequency of all social contacts on baseline day and day of challenge. We analyzed both the directed and undirected matrices as weighted networks to maintain the strength of the interactions, given our small per pen group size.

We examined direct and indirect social connections between the calves’ and their pen mates with three centrality measures at the individual or node level: degree, strength and eigenvector (reviewed in^27^). Nodal metrics describe each node’s position in the network relative to the other nodes^28^. These measures were chosen because they have proved useful in describing other dairy calves’ social networks^14^. Degree centrality examines the numbers of social connections that an individual node has. This measure indicates a node’s importance in the network and represents direct connections between individuals by measuring the number of edges that are connected to the node. The more nodes that a focal subject interacts with, the more central it is and the higher its degree centrality score. Strength, also known as weighted degree centrality, is a measure of the sum of weights assigned to the node's direct connections and represents the node strength^13,29^. Lastly, the eigenvector centrality is based on the sum of the centralities of an individual’s neighbors and the centrality of that node’s direct and indirect connections. For the directed networks of all-social contacts on challenge day, we also calculated the number of connections given and received (in-degree and out-degree) and the strength of these in-coming and out-going connections (in-strength and out-strength)^30^. Centrality measures were calculated using the *igraph* package in R (R Development Core Team, Version 4.1.1^31,32^) .

### Data analysis

To assess the effects of the disease challenge on social network position we examined both the directed and undirected networks. First, we generated separate linear mixed-effect models (LMMs) with the function “lmer” in the R package *lme4*^33^. Social network metrics derived from directed interactions on challenge day were entered as the dependent variable. In all models, we included treatment (MH/CON) and age as fixed effects and pen number as a random factor. Calves were housed in pens according to their age (mean ± SD: 46.5 ± 1.52 days in pen 1, 41.0 ± 3.2 days in pen 2, 29.7 ± 5.1 days in pen 3, and 26.5 ± 1.87 days in pen 4); therefore, these two variables were significantly positively correlated (r = 0.871, df = 24, *p* = 0.01, Pearson correlation test). To analyze the effects of the disease challenge for the 3 undirected networks (social lying frequency, social lying duration, and all-social contacts frequency), we calculated the differences in individual centrality scores (degree, strength, eigenvector) from baseline (Day −1) to challenge (Day 0). These differences, represented as positive or negative values, were entered as the predictor in separate LMMs, with treatment (MH/CON) and age as fixed effects and pen number as a random effect.

For each LMM, model assumptions (i.e., residual normality distribution plots) were visually checked using “check_model” from the R package *performance*^34^. Four variables were log_10_ transformed to achieve a normal distribution (directed networks: in-strength and out-strength, undirected networks: social lying frequency eigenvector difference and social lying duration strength difference). We tested for multicollinearity of the independent factors by calculating the variance inflation factor “vif” in R package *car*^35^. There was no evidence of multicollinearity between factors (maximum variance inflation factor = 1.01). All analyses were performed in R with two-tailed tests and alpha level set at 0.05^32^.

To account for the relational nature of social network data, permutation-based regression tests (e.g., node label permutations) have been widely adopted^36^. Hart et al.^37^ found that node permutations can be replaced with parametric regression given a well-specified parametric model when centrality metrics are regressed against nodal covariates. Although our models met this criterion, given our calves were sampled equally through automated observational methods and were restricted to their respective group-based pens, node permutations should also perform well^38^.

Our null hypothesis procedure consisted of building 1000 random networks using node label permutations on our social network centrality metrics (“perm.net.nl”), package *ANTs* in R^39^. Our null model maintained the variance structure in terms of network positions and thus the same distribution of node values in each social network. We shuffled SNA centrality metric data between individuals within their respective pen networks after the network had been inferred. We then ran our original LMMs with the permuted statistics (1000 permutations of each SNA metric) with a confidence interval set at 95% to obtain unbiased significance tests for the coefficients^40^.

Lastly, in order to explore the similarity of the three networks (social lying frequency, social lying duration and all-social contacts), we used a multiple regression quadratic assignment procedure with double semipartialling (MRQAP-DSP) and 1000 permutations in the *sna* R package^41^. We compared the duration and frequency matrices of dyadic lying behavior and whether social lying is related to all social contacts. MRQAP controls for autocorrelations in matrix regressions by using a Monte Carlo method of permutation to test significance^42,43^. For these models we only used undirected data from baseline day in order to minimize possible effects from the challenge.

## Results

### MH challenged calves less connected in directed networks on challenge day

**Figure 1:**
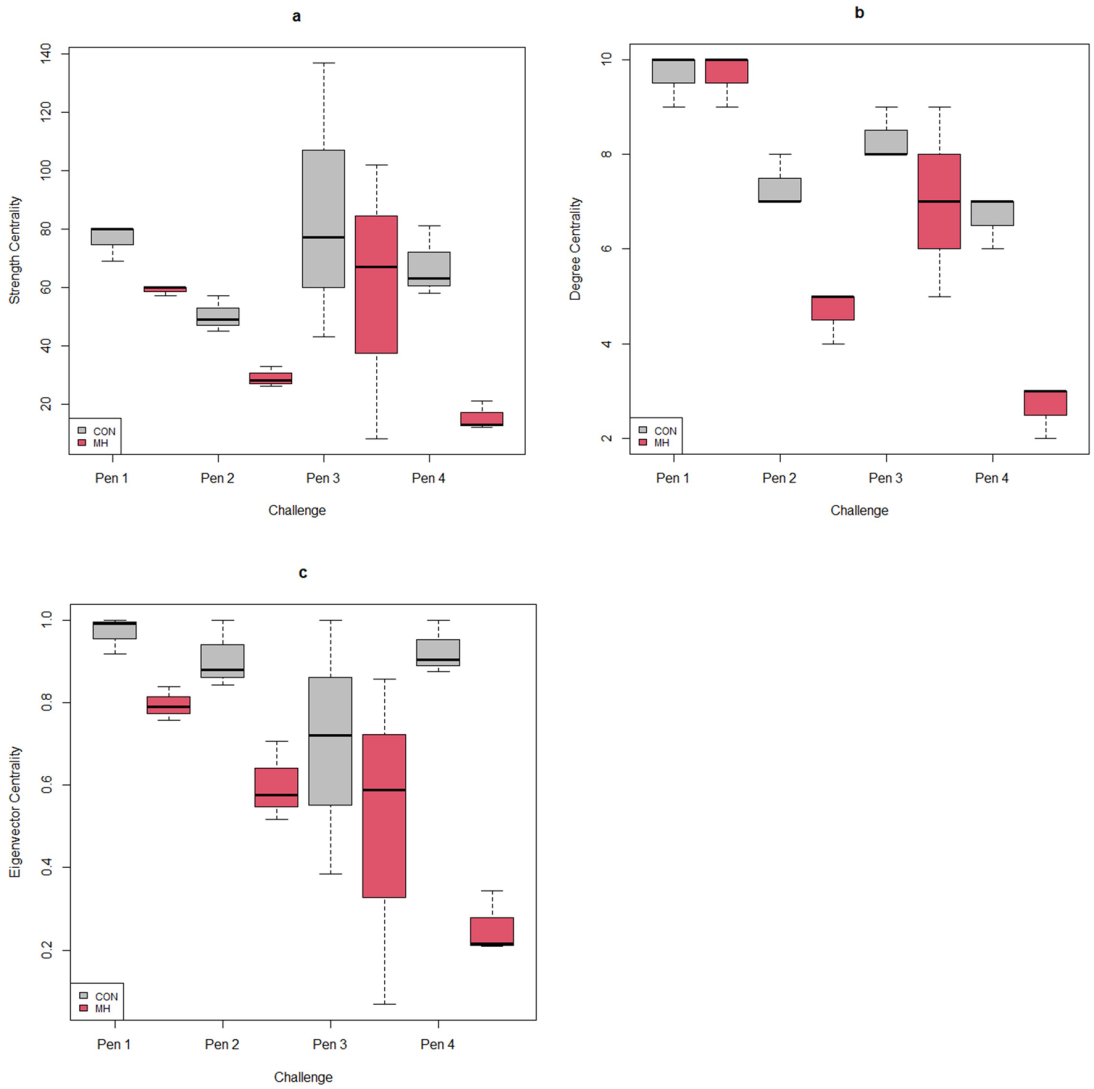
Diagnostic box plots represent the relationship between social network measures and an experimental challenge model (inoculation with *M. haemolytica)* or a sham procedure (inoculation with sterile saline) of bull calves (35.9 ± 8.8 d of age) housed in groups (6 calves/pen). Each plot shows the mean and distribution centrality network metrics (**a**) strength centrality, (**b**) degree centrality, (**c**) eigenvector centrality plotted for “challenged” (MH) and “healthy” (CON) individuals in each Pen (X-axis). Figure was created using R^32^.

We found strong evidence that the experimental challenge influenced the calves’ social network position on challenge day; challenged calves interacted with fewer penmates overall (lower degree centrality), spent less time in contact with others (lower strength centrality), and were less connected to more central individuals (lower eigenvector centrality) (Table 2; Fig. 1). Overall, control calves were more central and well-connected in their directed social contact network than would be expected by chance (*p* < 0.01 for eigenvector, degree and strength). Further, there was a significant, negative relationship between the experimental challenge and in-degree, in-strength network measures and a positive interaction between the challenge and out-degree. In other words, challenged calves initiated social contact with more penmates on challenge day (higher out-degree), while control calves received more social interactions from more penmates (higher in-strength/in-degree). Age was also associated with degree, out-degree and eigenvector centrality (Table 2).

**Table 2:** Results of linear mixed-effect models for directed networks of bull calves (35.9 ± 8.8 days of age) housed in groups (6 calves/pen): centrality metrics, testing the main fixed effects of calves exposed to an experimental challenge model (inoculation with *M. haemolytica)* or a sham procedure (inoculation with sterile saline) and age, random effect (Pen): coefficient estimates, standard errors, *t*-test and *p*-values.

Response variable	Fixed effects	Estimate ± SE	*z/t* Values	*p* values
Degree	Intercept	3.05 ± 2.36	1.29	0.22
	Challenge	− 2.11 ± 0.45	− 4.65	** < *0.01***
	Age	0.13 ± 0.06	2.25	***0.04***
In-degree	Intercept	2.31 ± 1.76	1.31	0.24
	Challenge	− 2.89 ± 0.51	− 5.58	** < *0.01***
	Age	0.07 ± 0.04	4.8	0.18
Out-degree	Intercept	0.59 ± 1.05	0.566	0.58
	Challenge	0.77 ± 0.18	4.08	** < *0.01***
	Age	0.06 ± 0.02	2.57	***0.02***
Eigenvector	Intercept	0.33 ± 0.16	2.03	0.054
	Challenge	− 0.34 ± 0.07	− 4.57	** < *0.01***
	Age	0.01 ± 0.01	3.44	** < *0.01***
Strength	Intercept	12.48 ± 39.12	0.31	0.76
	Challenge	− 30.68 ± 9.08	− 3.37	** < *0.01***
	Age	1.61 ± 1.04	1.54	0.17
In-strength*	Intercept	1.36 ± 0.62	2.17	0.08
	Challenge	− 0.68 ± 0.23	− 2.89	** < *0.01***
	Age	0.01 ± 0.02	0.29	0.77
Out-strength*	Intercept	1.16 ± 0.28	4.08	***0.02***
	Challenge	0.02 ± 0.09	0.28	0.78
	Age	0.01 ± 0.01	0.69	0.52

Significant values are in [bold].

*Denotes Log_10_ transformed variables.

### Reduced centrality in undirected networks for MH challenged calves during the challenge

**Figure 2:**
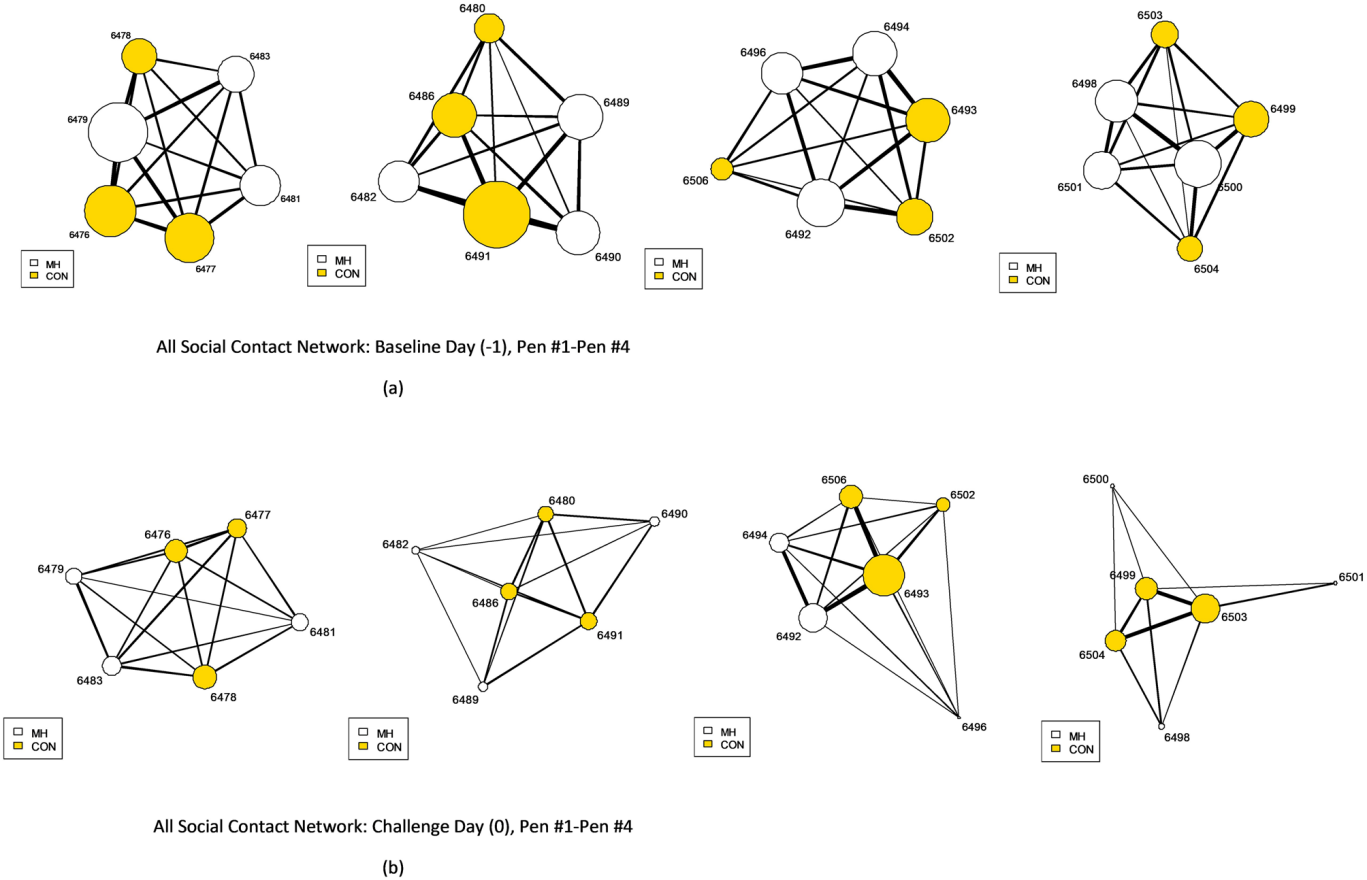
Network representation of the ‘All-Social Contacts Frequency’ networks of group-housed (*n* = 6/pen) bull calves (35.9 ± 8.8 d of age) exposed to an experimental challenge model (inoculation with *M. haemolytica)* or a sham procedure (inoculation with sterile saline): (**a**) baseline day: Pen#1–Pen#4, versus (**b**) challenge day: Pen#1–Pen#4. Individual calves (nodes) are represented by the circles; size is based on strength centrality; lines represent the weighted interactions among penmates. Baseline centrality scores were significantly different from centrality scores on challenge day and these differences were related to health. Figure was created using R^32^.

We calculated the change in network position to determine whether shifts in calves’ social connectedness were related to health. The difference in individual centrality scores, based on undirected interactions on baseline (Day -1) and challenge day (Day 0), were related to the experimental challenge for some measures (Table 3). In the all-social contacts network, the difference in scores for all three centrality measures were related to the challenge (*p* < 0.01 for eigenvector, degree and strength) (Table 3). Compared to control calves, challenged calves had a decrease in centrality and reduction in connectedness for all social contacts (Fig. 2). Conversely, control calves had an increase in the strength of social lying frequency connections. Changes in degree and eigenvector centrality for the frequency of social lying were not related to the challenge. Nor did we find relationships between the experimental challenge and changes in centrality for any measures of social lying duration.

**Table 3:** Results of linear mixed-effect models for undirected networks of bull calves (35.9 ± 8.8 days of age) housed in groups (6 calves/pen): differences in centrality measures between baseline and challenge day, testing the main fixed effects of calves exposed to an experimental challenge model (inoculation with *M. haemolytica)* or a sham procedure (inoculation with sterile saline) and age, random effect (Pen): coefficient estimates, standard errors, *t*-test and *p*-values.

Undirected network	Response variable	Fixed effects	Estimate ± SE	*z/t* values	*p* values
Social lying frequency	Eigenvector differences*	Intercept	− 0.21 ± 0.17	− 1.22	0.22
		Challenge	0.12 ± 0.07	1.59	0.13
		Age	− 0.01 ± 0.01	− 1.6	0.12
	Strength differences	Intercept	− 5.79 ± 12.09	− 0.48	0.64
		Challenge	6.26 ± 2.41	2.5	***0.02***
		Age	0.08 ± 0.31	0.25	0.8
	Degree differences	Intercept	− 1.07 ± 1.91	− 0.56	0.59
		Challenge	0.81 ± 0.45	1.78	0.09
		Age	0.02 ± 0.05	0.45	0.66
Social lying duration	Eigenvector differences	Intercept	− 0.12 ± 0.42	− 0.28	0.77
		Challenge	− 0.07 ± 0.19	− 0.39	0.69
		Age	0.01 ± 0.01	0.33	0.74
	Strength differences*	Intercept	3.58 ± 0.37	9.67	** < *0.01***
		Challenge	0.15 ± 0.18	0.79	0.43
		Age	0.02 ± 0.01	1.97	0.07
	Degree differences	Intercept	1.07 ± 1.91	7.04	0.59
		Challenge	− 0.81 ± 0.45	− 1.78	0.09
		Age	− 0.02 ± 0.05	− 0.45	0.66
All social contacts frequency	Eigenvector differences	Intercept	− 0.47 ± 0.22	− 2.10	0.05
		Challenge	− 0.47 ± 0.10	− 4.57	** < *0.01***
		Age	0.01 ± 0.01	2.85	** < *0.01***
	Strength differences	Intercept	− 20.09 ± 43.07	− 3.01	0.67
		Challenge	− 52.90 ± 17.82	− 18.86	** < *0.01***
		Age	1.11 ± 1.15	2.81	0.41
	Degree differences	Intercept	− 1.52 ± 0.95	− 1.60	0.15
		Challenge	− 0.70 ± 0.22	− 3.09	** < *0.01***
		Age	− 0.04 ± 0.02	− 1.59	0.15

Significant values are in [bold].

*Denotes Log_10_ transformed variables.

### Dyadic interactions differ for active social behavior and social rest

We evaluated the similarity of the duration and frequency of dyadic lying behavior during the baseline period and whether it is related to all social contacts among calves, to explore whether the three interaction networks show meaningful differences and if some measures are more useful in determining sickness behavior. We found that social lying frequency was a strong predictor of lying duration for all four of the pens on baseline day (MRQAP, pen1: r = 0.42, *p* = 0.01; pen2: r = 0.77, *p* < 0.001; pen3: r = 0.78, *p* < 0.001; pen4: r = 0.83, *p* < 0.001). Notably, neither lying frequency nor duration predicted affiliative interactions in the all-social contact networks. This suggests that calves’ affiliative interactions are different for active social interactions (e.g., grooming and head butting), compared to more passive social rest.

## Discussion

Our study assessed the effects of an experimental challenge on the social networks of group-housed dairy calves. To our knowledge, this is the first study to examine individual centrality (connectedness within the social network) of dairy calves following experimental challenge of a respiratory pathogen. As predicted, we found challenged calves (compared to controls) were less central in their directed social contact networks on challenge day. In addition, challenged calves but not controls, had reduced centralities for all three measures in the undirected social contact network, baseline to challenge day. These results are consistent with previous work on social network position which found that immune-challenged bats injected with lipopolysaccharide had lower individual centrality scores in association networks, compared to healthy bats^2^. Further, it adds to a growing body of evidence that sickness behavior^44,45^ includes a reduction in social connections and social preference with conspecifics and is indicative of illness in a variety of species (e.g., mammals:^46^, fish:^47^, insects:^48^). In contrast, control calves had on average higher levels of centrality for directed social contact networks, and an increase in strength centrality for undirected social lying frequency networks, from baseline to challenge day. This suggests healthy calves did not avoid challenged penmates, and instead increased social lying connectedness. Although it may be advantageous to avoid infectious conspecifics^49^, increased social interaction with sick penmates has also been reported in group-housed pigs^50,51^.

Illness and inflammation in humans have been linked with an increase in approach behavior towards close contacts^7^, who may offer support and care to aid in recovery^52^. These findings lend support to the effect we found on higher social contact out-degree for challenged calves. It may be that initiating social contact is more beneficial than receiving social interactions when an individual is sick. Grooming is an important affiliative behavior that primarily functions to maintain social bonds in many social species (e.g.^53^), including calves^15^, (reviewed in^54^). In nonhuman primates, giving rather than receiving grooming has been shown to mitigate social stress and stress-related hormone levels (*Macaca sylvanus*:^55^; *Papio cynocephalus ursinus:*^56^). While animals housed in pens cannot completely withdraw from infected group members, our results indicate calves exhibit more complex sickness behavior by increasing some social interactions with familiar peers, similar to that seen in humans and nonhuman primates (reviewed in^8,10^).

There is considerable interest in measuring behavior as an indicator of disease in dairy calves, given the long-term animal welfare consequences. In particular, bovine respiratory disease is the second leading cause of early life mortality in preweaned dairy calves^57^. Previous work has evaluated how changes in behavior may coincide with respiratory disease in dairy calves, either naturally occurring or resulting from an experimental disease challenge. For example, disease has been associated with changes in patterns of milk feeding (reviewed in^58^) and lying behavior (reviewed in^59^). Calves with naturally occurring respiratory disease have shown a decrease in the frequency of lying bouts (e.g.^60^) and a reduction in milk feeding behaviors (e.g.^61^), compared to healthy calves. Our study expands on these findings and suggests that in addition to changes in feeding and activity behavior, calves modify their social interactions in response to experimentally induced illness.

We examined social rest given evidence that this may be a meaningful indicator of social bonding^62^. In group-housed dairy calves, greater durations of social rest were previously observed between familiar calves, compared to unfamiliar calves^21^. While an increase in lying time^60^ and the frequency of lying bouts^63^ have been associated with naturally occurring respiratory disease, we found no evidence that changes in individual centrality for social lying duration were associated with sickness. Nor did we find relationships between the changes in individual degree and eigenvector centrality for social lying frequency and the disease challenge for either challenged or control calves. Calf space allowance was somewhat greater than industry standard for dairy calves (e.g. 4.6 m^2^/calf:^64^) in this study, but not outside the range of observed values. Low space allowance has been linked to a reduction in play behavior in diary calves^65^, while an increase in allowance is associated with reduced lying behavior^66^. However, to our knowledge, research on the effects of space allowance and the expression of social behavior has only been conducted on healthy individuals.

In order to evaluate the potential use of changes in relationship between social network position and its relation to health, it is important to distinguish between networks of discrete behaviors. We found that the social lying frequency network was a strong predictor of the social lying duration network. However, neither social lying frequency nor duration predicted the all-social contact network. These results suggest that network measures of directed affiliative interactions (e.g. social grooming and head butting) are different than network measures of passive social rest (e.g. frequency and duration of social lying bouts). Thus, networks constructed with active and passive affiliative interactions may be useful in understanding changes in social interactions and sickness behavior.

## Conclusion

Here, we provide evidence that an experimental challenge influenced the social networks of group-housed dairy calves. Compared to control calves, challenged calves had reduced centrality and social connectedness over the two-day challenge and less central network positions on challenge day. Additionally, challenged calves initiated contact with more penmates on challenge day, potentially functioning to elicit social support. In addition to previous findings that focused on initiated dyadic social interactions, this is the first study demonstrating that changes in social network position coincide with an experimentally induced challenge in calves.

## Data Availability

The data that support the findings of this study are available from the corresponding author, E.K.M.C., upon request.

## References

[1] Dubrovsky SA (2019). Bovine respiratory disease (BRD) cause-specific and overall mortality in preweaned calves on California dairies: The BRD 10K study. J. Dairy Sci..

[2] Ripperger SP, Stockmaier S, Carter GG (2020). Tracking sickness effects on social encounters via continuous proximity sensing in wild vampire bats. Behav. Ecol..

[3] Hamilton DG (2020). Infectious disease and sickness behaviour: tumour progression affects interaction patterns and social network structure in wild Tasmanian devils. Proc. Biol. Sci..

[4] Dantzer R (2009). Cytokine, sickness behavior, and depression. Immunol. Allergy Clin. N. Am..

[5] Lopes PC, Block P, König B (2016). Infection-induced behavioural changes reduce connectivity and the potential for disease spread in wild mice contact networks. Sci. Rep..

[6] Willette AA, Lubach GR, Coe CL (2007). Environmental context differentially affects behavioral, leukocyte, cortisol, and interleukin-6 responses to low doses of endotoxin in the rhesus monkey. Brain Behav. Immun..

[7] Inagaki TK (2015). The role of the ventral striatum in inflammatory-induced approach toward support figures. Brain Behav. Immun..

[8] Eisenberger NI, Inagaki TK, Mashal NM, Irwin MR (2010). Inflammation and social experience: an inflammatory challenge induces feelings of social disconnection in addition to depressed mood. Brain Behav. Immun..

[9] Miller AH, Maletic V, Raison CL (2009). Inflammation and its discontents: the role of cytokines in the pathophysiology of major depression. Biol. Psych..

[10] Shattuck EC, Muehlenbein MP (2015). Human sickness behavior: ultimate and proximate explanations. Am. J. Phys. Anthropol..

[11] Hixson CL, Krawczel PD, Caldwell JM, Miller-Cushon EK (2018). Behavioral changes in group-housed dairy calves infected with Mannheimia haemolytica. J. Dairy Sci..

[12] Cantor MC, Costa JHC (2022). Daily behavioral measures recorded by precision technology devices may indicate bovine respiratory disease status in preweaned dairy calves. J. Dairy Sci..

[13] Croft, D. P., James, R. & Krause, J. *Exploring animal social networks*. (Princeton University Press, Princeton, 2008). doi:10.1515/9781400837762.

[14] Bolt SL, Boyland NK, Mlynski DT, James R, Croft DP (2017). Pair housing of dairy calves and age at pairing: effects on weaning stress, health, production and social networks. PLoS ONE.

[15] de Freslon I, Martínez-López B, Belkhiria J, Strappini A, Monti G (2019). Use of social network analysis to improve the understanding of social behaviour in dairy cattle and its impact on disease transmission. Appl. Anim. Behav. Sci..

[16] Lecorps B, Kappel S, Weary DM, von Keyserlingk MAG (2019). Social proximity in dairy calves is affected by differences in pessimism. PLoS ONE.

[17] Val-Laillet D, Guesdon V, von Keyserlingk MAG, de Passillé AM, Rushen J (2009). Allogrooming in cattle: Relationships between social preferences, feeding displacements and social dominance. Appl. Anim. Behav. Sci..

[18] Foris B, Zebunke M, Langbein J, Melzer N (2019). Comprehensive analysis of affiliative and agonistic social networks in lactating dairy cattle groups. Appl. Anim. Behav. Sci..

[19] Chua B, Coenen E, Weary DM (2002). Effects of pair versus individual housing on the behavior and performance of dairy calves. J. Dairy Sci..

[20] Horvath KC, Miller-Cushon EK (2019). Evaluating effects of providing hay on behavioral development and performance of group-housed dairy calves. J. Dairy Sci..

[21] Miller-Cushon EK, DeVries TJ (2016). Effect of social housing on the development of feeding behavior and social feeding preferences of dairy calves. J. Dairy Sci..

[22] Færevik G, Andersen IL, Jensen MB, Bøe KE (2007). Increased group size reduces conflicts and strengthens the preference for familiar group mates after regrouping of weaned dairy calves (*Bos taurus*). Appl. Anim. Behav. Sci..

[23] Griffin D, Chengappa MM, Kuszak J, McVey DS (2010). Bacterial pathogens of the bovine respiratory disease complex. Vet. Clin. N. Am. Food. Anim. Pract..

[24] XXX. https://www.aphis.usda.gov/animal_health/nahms/dairy/downloads/dairy14/Dairy14_dr_PartIII.pdf. https://www.aphis.usda.gov/animal_health/nahms/dairy/downloads/dairy14/Dairy14_dr_PartIII.pdf.

[25] Eberhart NL, Storer JM, Caldwell M, Saxton AM, Krawczel PD (2017). Behavioral and physiologic changes in Holstein steers experimentally infected with Mannheimia haemolytica. Am. J. Vet. Res..

[26] Friard O, Gamba M (2016). BORIS: a free, versatile open-source event-logging software for video/audio coding and live observations. Methods Ecol. Evol..

[27] Sosa S, Sueur C, Puga-Gonzalez I (2021). Network measures in animal social network analysis: Their strengths, limits, interpretations and uses. Methods Ecol. Evol..

[28] Butts CT (2008). Social network analysis: A methodological introduction. Asian J. Soc. Psychol..

[29] Hanneman, R. A. & Riddle, M. A brief introduction to analyzing social network data. in *The SAGE handbook of social network analysis* 331–339 (SAGE Publications Ltd, New York, 2014). doi:10.4135/9781446294413.n23.

[30] Newman MEJ (2004). Analysis of weighted networks. Phys. Rev. E Stat. Nonlin. Soft Matter Phys..

[31] Igraph: Network analysis software. https://igraph.org.

[32] R: The R Project for Statistical Computing. https://www.R-project.org/.

[33] Bates D, Mächler M, Bolker B, Walker S (2015). Fitting linear mixed-effects models using lme4. J. Stat. Softw..

[34] Lüdecke D, Ben-Shachar M, Patil I, Waggoner P, Makowski D (2021). Performance: an R package for assessment, comparison and testing of statistical models. JOSS.

[35] R-Forge: car: Companion to Applied Regression: Project Home. https://r-forge.r-project.org/projects/car/.

[36] Croft DP, Madden JR, Franks DW, James R (2011). Hypothesis testing in animal social networks. Trends Ecol. Evol..

[37] Hart JDA, Franks DW, Brent LJN, Weiss MN (2021). Accuracy and power analysis of social networks built from count data. Methods Ecol. Evol..

[38] Farine DR, Carter GG (2021). Permutation tests for hypothesis testing with animal social network data: Problems and potential solutions. Methods Ecol. Evol..

[39] Sosa S (2020). A multilevel statistical toolkit to study animal social networks: the animal network toolkit software (ANTs) R package. Sci. Rep..

[40] Farine DR (2017). A guide to null models for animal social network analysis. Methods Ecol. Evol..

[41] Butts CT (2008). Social network analysis with sna. J. Stat. Softw..

[42] Dekker D, Krackhardt D, Snijders TAB (2007). Sensitivity of MRQAP tests to collinearity and autocorrelation conditions. Psychometrika.

[43] Krackhardt D (1988). Predicting with networks: nonparametric multiple regression analysis of dyadic data. Soc. Netw..

[44] Kent S, Bluthé RM, Kelley KW, Dantzer R (1992). Sickness behavior as a new target for drug development. Trends Pharmacol. Sci..

[45] Kelley KW (2003). Cytokine-induced sickness behavior. Brain Behav. Immun..

[46] Stockmaier S, Bolnick DI, Page RA, Carter GG (2018). An immune challenge reduces social grooming in vampire bats. Animal Behav..

[47] Kirsten K, Soares SM, Koakoski G, Carlos Kreutz L, Barcellos LJG (2018). Characterization of sickness behavior in zebrafish. Brain. Behav. Immun..

[48] Kazlauskas N, Klappenbach M, Depino AM, Locatelli FF (2016). Sickness behavior in honey bees. Front. Physiol..

[49] Klein SL (2000). The effects of hormones on sex differences in infection: from genes to behavior. Neurosci. Biobehav. Rev..

[50] Munsterhjelm C (2019). Sick and grumpy: Changes in social behaviour after a controlled immune stimulation in group-housed gilts. Physiol. Behav..

[51] Veit C (2021). The use of social network analysis to describe the effect of immune activation on group dynamics in pigs. Animal.

[52] Muscatell KA (2016). Exposure to an inflammatory challenge enhances neural sensitivity to negative and positive social feedback. Brain Behav. Immun..

[53] Schino G (2001). Grooming, competition and social rank among female primates: a meta-analysis. Animal Behav..

[54] Costa JHC, Cantor MC, Adderley NA, Neave HW (2019). Key animal welfare issues in commercially raised dairy calves: social environment, nutrition, and painful procedures. Can. J. Anim. Sci..

[55] Shutt K, MacLarnon A, Heistermann M, Semple S (2007). Grooming in Barbary macaques: better to give than to receive?. Biol. Lett..

[56] Barrett L, Henzi SP, Weingrill T, Lycett JE, Hill RA (1999). Market forces predict grooming reciprocity in female baboons. Proc. R. Soc. Lond. B.

[57] Dubrovsky SA (2020). Preweaning cost of bovine respiratory disease (BRD) and cost-benefit of implementation of preventative measures in calves on California dairies: The BRD 10K study. J. Dairy Sci..

[58] Morrison J (2021). Predicting morbidity and mortality using automated milk feeders: A scoping review. J. Dairy Sci..

[59] Costa JHC, Cantor MC, Neave HW (2021). Symposium review: Precision technologies for dairy calves and management applications. J. Dairy Sci..

[60] Swartz TH, Findlay AN, Petersson-Wolfe CS (2017). Short communication: Automated detection of behavioral changes from respiratory disease in pre-weaned calves. J. Dairy Sci..

[61] Borderas TF, Rushen J, von Keyserlingk MAG, de Passillé AMB (2009). Automated measurement of changes in feeding behavior of milk-fed calves associated with illness. J. Dairy Sci..

[62] Sato S, Wood-Gush DG (1987). Observations on creche behaviour in suckler calves. Behav. Process..

[63] Duthie CA (2021). Feeding behaviour and activity as early indicators of disease in pre-weaned dairy calves. Animal.

[64] Jorgensen MW (2017). Factors associated with dairy calf health in automated feeding systems in the Upper Midwest United States. J. Dairy Sci..

[65] Jensen MB, Vestergaard KS, Krohn CC (1998). Play behaviour in dairy calves kept in pens: the effect of social contact and space allowance. Appl. Anim. Behav. Sci..

[66] Sutherland MA, Worth GM, Stewart M (2014). The effect of rearing substrate and space allowance on the behavior and physiology of dairy calves. J. Dairy Sci..

